# Multimodal natural language processing in ophthalmology: bridging clinical text and medical imaging

**DOI:** 10.3389/fmed.2026.1762586

**Published:** 2026-07-28

**Authors:** Shuyan Qin, Nana Zhao, Lei Shen, Yanan Zhang, Shengfang Ge

**Affiliations:** 1 Department of Ophthalmology, Nanjing Drum Tower Hospital Group Suqian Hospital, Suqian Hospital Affiliated to Xuzhou Medical University, Suqian, China; 2 Department of Ophthalmology, Ninth People’s Hospital, Shanghai Jiao Tong University School of Medicine, Shanghai, China; 3 Shanghai Key Laboratory of Orbital Diseases and Ocular Oncology, Shanghai, China

**Keywords:** artificial intelligence, ChatGPT, multimodal, natural language processing, ophthalmology

## Abstract

Multimodal natural language processing (NLP) represents a transformative approach in ophthalmology by bridging clinical text and medical imaging to enhance diagnostic accuracy, workflow efficiency, and patient-centered care. The mainstream NLP technologies were applied in ophthalmology, such as self-attention and cross-modality attention in multimodal settings, BERT and GPT in text-focused settings, and vision-focused transformers in imaging-focused settings. The applications of multimodal NLP in ophthalmology cover three key domains as follows: clinical text-image integration for comprehensive data analysis, enhanced screening and diagnostic prediction systems, and improved patient communication and management. With the significant advancements of multimodal NLP applications in ophthalmology, the consequent challenges must be addressed, including data privacy concerns, domain-specific terminology adaptation, model interpretability, standardization of multimodal data, and regulatory validation. Emerging directions (e.g., few-shot learning, real-time interactive systems, and privacy-preserving federated learning) offer potential solutions to current challenges. The successful implementation of multimodal NLP in ophthalmology requires collaborative efforts to develop standardized datasets, refine ethical guidelines, and validate clinical utility. By synthesizing textual and visual data, these technologies are poised to reshape ophthalmic practice, ultimately leading to more precise, accessible, and personalized eye care.

## 1 Introduction

Artificial intelligence (AI) has transformed modern medicine, with natural language processing (NLP) emerging as a pivotal innovation (1, 2). Powered by transformer-based architectures, contemporary NLP systems excel in contextual comprehension, long-range dependency analysis, and cross-linguistic adaptation (3). These capabilities allow NLP to interpret intricate clinical documentation, automate medical record-keeping, and support diagnostic decision-making. The tendency of NLP application in clinical medicine is unstoppable, particularly for applications such as electronic health record (EHR) parsing, clinician-patient communication, and diagnostic assistance (4). Among them, ophthalmology demands both precision and the ability to synthesize heterogeneous data, so it especially calls on the NLP application (5).

Ophthalmic practice fundamentally relies on multimodal data integration, combining unstructured clinical narratives with complex imaging modalities. The significance of clinical notes in ophthalmology is distinct and critical, as they capture subjective patient experiences, dynamic symptoms, and longitudinal context that static structural findings cannot provide. The reports offer rich textual context, enabling models to learn long-tailed representations with hierarchical concepts and better simulate the cognitive processes of ophthalmologists in complex clinical scenarios. Furthermore, while structured data requires labor-intensive conversion prone to information loss, unstructured notes preserve the full richness of clinical nuance (6). Fundamentally, ophthalmic imaging is particularly unique, involving specialized modalities such as optical coherence tomography, fundus photography, and ocular ultrasound, which require precise interpretation within this broader clinical context (7).

The necessity for multimodal synthesis is explicitly codified in major clinical guidelines in ophthalmology. For instance, Diabetic Retinopathy Preferred Practice Pattern® for diabetic retinopathy (DR) emphasized that diagnosis and management decisions must integrate patient history such as duration of diabetes, past glycemic control, and ocular history, and examinations such as visual acuity, intraocular pressure (IOP), gonioscopy, and fundoscopy (8). Similarly, guidelines for glaucoma management mandate the correlation of imaging such as OCT disc imaging with functional visual field tests and patient-reported symptoms, to distinguish between true progression and measurement variability according to European Glaucoma Society (9). These standards highlight that text (patient history, clinical metrics) and imaging are not isolated data sources but complementary pillars of diagnostic reasoning. Consequently, NLP systems in ophthalmology must inherently adopt a multimodal approach to align with the evidence-based practices.

The inherent variability of these data types, coupled with domain-specific terminology and dynamic anatomical relationships, challenges conventional unimodal analysis methods (10, 11). While text-based systems may miss critical imaging correlations, and purely visual approaches often lack clinical context, multimodal AI techniques, especially novel NLP, offer a solution by bridging these clinical text and medical imaging.

NLP serves as a cornerstone for multimodal integration through its ability to establish cross-modal relationships, such as linking textual descriptions of retinal pathology with corresponding imaging biomarkers (12). Advanced architectures like Contrastive Language-Image Pretraining (CLIP) and Biomedical Vision-Language (BioMedVL) demonstrate this potential by jointly encoding visual and textual ophthalmic data, enabling applications ranging from semantic image retrieval to automated consistency verification between radiology reports and scan interpretations (13, 14). Such capabilities are indispensable in clinical ophthalmology (15, 16), where diagnostic accuracy hinges on synthesizing narrative observations with detailed imaging findings. By harmonizing these modalities, NLP-driven systems promise to elevate diagnostic precision, optimize clinical workflows, and advance personalized patient management in ophthalmology.

## 2 The mainstream natural language processing technologies

**Figure 1:**
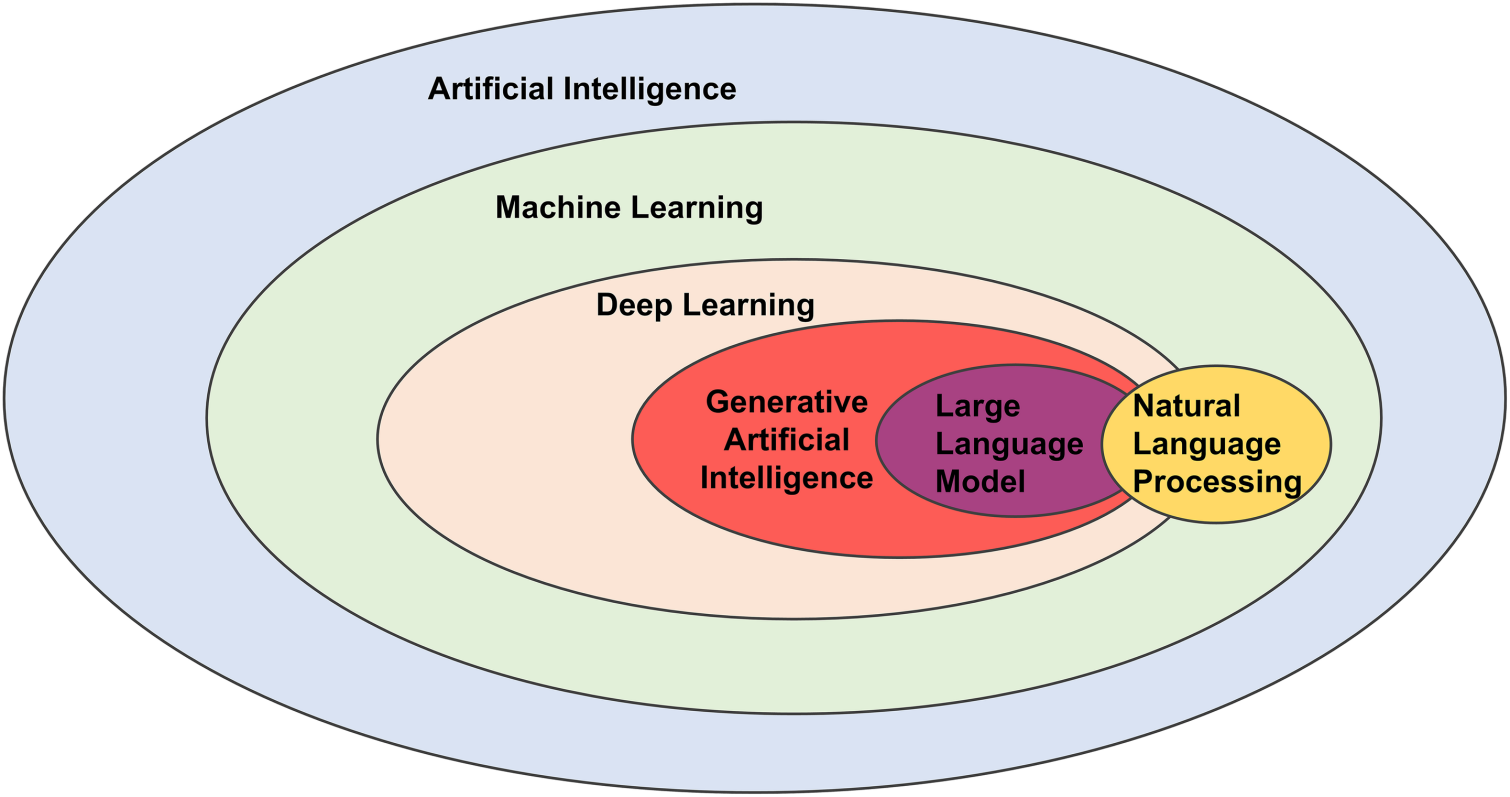
Current commonly used artificial intelligence technologies and the interrelationships. The current commonly utilized artificial intelligence technologies cover machine learning, deep learning, generative artificial intelligence, large language models, and natural language processing.

AI is revolutionizing healthcare by enabling machines to perform tasks traditionally requiring human cognition. Within AI, deep learning (DL) has emerged as a dominant paradigm, leveraging neural networks to extract patterns from complex data (17–20). The rising technology, NLP, focuses on enabling machines to understand, interpret, and generate human language, while large language models (LLMs) and generative AI (GAI) represent its cutting edge, powered by transformer architectures (21, 22). Transformers, with their self-attention mechanisms, have become the backbone of modern NLP (23, 24), facilitating breakthroughs in multimodal applications. These advancements underpin the success of multimodal NLP in ophthalmology, where integrating heterogeneous data is critical for accurate diagnosis and decision-making (Figure 1).

The success of multimodal NLP in ophthalmology relies on advanced architectures capable of processing and aligning heterogeneous data (25). Self-attention and cross-modality attention mechanisms serve as the foundation for modeling complex relationships between text and images (26, 27). Self-attention enables the model to dynamically weigh the importance of each word or image region within a sequence, capturing long-range dependencies (28). Cross-modality attention further extends this capability by establishing explicit interactions between textual tokens and visual features (29). The bidirectional alignment is critical for tasks like automated report generation or discrepancy detection, where contextual coherence across modalities determines clinical utility.

Text-focused models have evolved significantly, moving from traditional sequence modeling to advanced generative architectures. While earlier models like Word2Vec and long short-term memory (LSTM) laid the groundwork, the advent of LLMs has transformed clinical text analysis. BERT and its biomedical variants including BioBERT and ClinicalBERT, excel in understanding ophthalmic narratives (30–32). BERT’ s bidirectional training objective enables deep understanding of medical jargon, making it ideal for EHR classification or entity extraction (33). The recent advance in LLMs, particularly the GPT family such as GPT-4 and LLaMA, has introduced autoregressive generative capabilities that go beyond classification. The models can synthesize comprehensive diagnostic reports from fragmented clinical inputs, generate patient-friendly summaries, and perform complex reasoning tasks such as differential diagnosis generation (34). Fine-tuned ophthalmic LLMs, such as OphthaBERT and EyeLLM, further optimize performance by incorporating domain-specific pretraining on ophthalmology literature, significantly reducing hallucinations and improving adherence to clinical guidelines. However, these models lack native image-processing capabilities, necessitating integration with vision architectures for multimodal tasks.

To complement text-processing models, robust vision architectures are essential. Historically, deep convolutional neural networks (CNNs) such as ResNet, VGG, and EfficientNet were the gold standard for ophthalmic image analysis due to their ability to capture local spatial hierarchies and translation invariance (35). CNNs remain widely used for tasks like DR screening and glaucoma detection due to their computational efficiency and proven reliability (36, 37). However, the emergence of Vision Transformers (ViT) has revolutionized the field by applying self-attention mechanisms to image patches, allowing models to capture global contextual relationships that CNNs may miss (38–40) Recent adaptations like RETFound pretrain ViT on massive retinal image datasets to generalize across diseases, while models such as OCT-Transformer specialize in layer-wise segmentation of OCT volumes (41). Furthermore, generative vision models, such as diffusion models and generative adversarial networks (GANs), are increasingly used for image-to-image translation, data augmentation, and synthesizing high-quality medical images to address data scarcity (42–44). Despite the strengths, pure vision models struggle with textual context, underscoring the need for multimodal extensions.

Multimodal models bridge the gap by jointly encoding text and images. CLIP aligns visual and linguistic representations through contrastive learning, enabling tasks like image retrieval via natural language queries (45–47). Its medical counterpart, BioMedCLIP, enhances this by training on paired radiology reports and images, adapting to ophthalmic data with fine-tuning (14, 48, 49). Fusion of Language and Image Representations (FUSION) advances further by incorporating EHR context (50, 51). These models unlock end-to-end applications, from generating differential diagnoses to validating imaging findings against clinical notes, thereby closing the loop between ophthalmic data modalities.

## 3 The applications of multimodal NLP in ophthalmology

**Figure 2:**
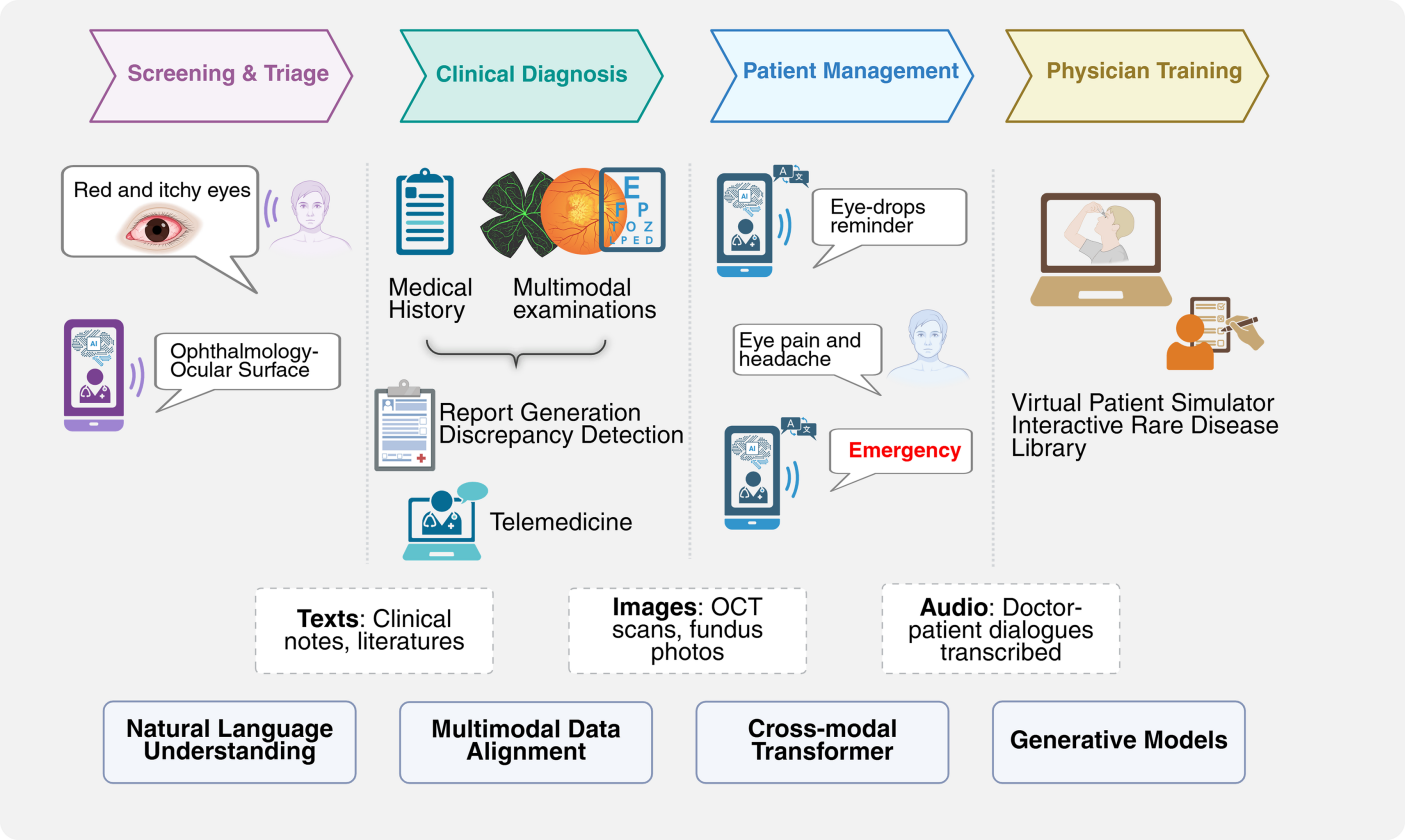
The applications of natural language processing in ophthalmology. The main settings include screening and triage, clinical diagnosis, patient management, and physician training scenarios. The multimodal data covers texts (clinical notes and literature), images (OCT scans and fundus photos), and audio (transcribed doctor-patient dialogs), with key enabling technologies such as natural language understanding, multimodal data alignment, cross-modal transformers, and generative models. Created with BioRender.com.

The clinical implementation of multimodal NLP in ophthalmology manifests across the following interdependent directions, each addressing distinct challenges in data synthesis and interpretation (Figure 2). These applications collectively demonstrate how language-vision integration transcends conventional AI limitations in ophthalmic practice.

### 3.1 Clinical text-image integration

Automatic report generation is a significant task as it can alleviate the workload of physicians and minimize regional discrepances in medical settings (52). However, it’s challenging because the computational model needs to mimic physicians to obtain multimodal information, including clinical information, medical images, and medical knowledge, and then produce accurate and comprehensive reports (53). Nowadays, modern NLP systems can transform clinical raw data into structured diagnostic reports by learning latent mappings between pixel-level features and clinical semantics. The FFA-GPT (54), an automated pipeline, and the InterpreFFA (55), a diagnosis-supervised contrastive learning framework, were demonstrated the ability to generate structured reports, achieving clinician-approved accuracy. Contrarily, a text-to-video generative AI model was constructed for converting FFA report texts into dynamic FFA videos, showing its potential in clinical or educational visualization and addressing medical data sharing restrictions (56). This capability is further advanced by foundation models like EyeCLIP (14), which demonstrated how multimodal pretraining on 2.77 million ophthalmic images and clinical texts can enhance cross-modal alignment. EyeCLIP’s novel training strategy, combining self-supervised reconstruction with image-text contrastive learning, enables robust performance in disease classification, visual question answering (VQA), and zero-shot scenarios, addressing long-tail distribution challenges in rare ophthalmic conditions.

Crucially, clinical variables serve as a vital semantic bridge between text and imaging modalities. The variables, such as patient demographics, vision acuity, and IOP, may be recorded in narrative notes or estimated directly from image data. For instance, in school-level myopia management, predictive models leveraging clinical variables extracted from records, such as age, vision acuity, refraction, showed potential in forecasting myopia onset and progression, demonstrating how structured textual inputs can anchor predictive models (57). Besides, in glaucoma diagnosis, multimodal approaches such as the GAMMA Challenge integrate fundus images and OCT volumes to estimate key clinical biomarkers like the vertical cup-to-disc ratio and retinal nerve fiber layer thickness directly from imaging data (58). Furthermore, studies on glaucoma progression showed that supplementing longitudinal visual field data with clinical variables including central corneal thickness and IOP, significantly improved predictive accuracy over imaging-only models (59).

Beyond report generation, the semantic alignment of imaging databases with clinical language enables granular cross-modal search (60, 61). By encoding both radiology lexicons and visual features into a shared latent space, NLP systems allow clinicians to practice image-text retrieval, text-image retrieval, and even image-image retrieval. Models like EyeCLIP exemplify this potential, having shown superior performance in cross-modal retrieval tasks compared to general-domain or medical-domain counterparts. The cross-modal retrieval significantly enriches the search paradigm, accelerates clinical decision support and research cohort building.

Further, NLP can be integrated with Picture Archiving and Communication Systems (PACS) by bridging clinical texts with medical imaging (62). This synergy enables semantic image retrieval, allowing physicians to query PACS using natural language descriptions rather than relying solely on structured DICOM metadata. Emerging federated learning frameworks extend this synergy across healthcare networks, as evidenced by EyeCLIP’s training across diverse Chinese hospitals, improving model generalizability while maintaining data privacy. These advancements collectively empower standardized, large-scale ophthalmic AI systems, transforming diagnostic workflows from documentation to decision support (63).

### 3.2 Enhanced screenings, diagnostics and predictions

Multimodal NLP demonstrates significant potential in revolutionizing ophthalmic screening and diagnostic workflows by integrating clinical narratives with medical imaging. While early implementations like GPT-4 V show promise in basic tasks (e.g., 95.6% accuracy in imaging modality identification), they reveal critical limitations in clinical settings, achieving only 42% diagnostic accuracy for slit-lamp images and 13.7% for fundus photography, with 35% of correct diagnoses supported by flawed rationales (64). However, rigorously validated multimodal NLP systems are progressing. A prime example is DeepDR-LLM (65), an integrated image-language system that synergizes deep learning-based retinal analysis with LLMs to improve DR screening in primary care settings. In clinical validations, the system demonstrated significant efficacy: when assisting primary care physicians (PCPs), the accuracy for identifying referable DR increased from 81.0% (unassisted) to 92.3% (assisted). Recently, the team developed EyeFM (65), an updated multimodal vision-language eyecare system, and conducted a comprehensive evaluation, including retrospective validations, multicountry efficacy validation as a clinical copilot and a double-masked randomized controlled trial (RCT). The study specifically targeted the screening and diagnosis of retinal diseases within a high-risk population, focusing on conditions such as DR, glaucoma, and age-related macular degeneration. The research firstly provided high-quality clinical evidence to support the implementation of emerging NLP into real-world clinical scenarios for clinical assistance. In the RCT involving 668 participants, ophthalmologists assisted by the EyeFM copilot achieved a significantly higher correct diagnostic rate (92.2% vs. 75.4%, *p* < 0.001) and referral rate (92.2% vs. 80.5%, *p* < 0.001) compared to the standard care group. Furthermore, the system improved the standardization of clinical reports (median score 37 vs. 33, *p* < 0.001).

The multimodal NLP can streamline the clinical workflows by automatically correlating imaging findings with relevant clinical documentation, generating preliminary diagnostic reports that reduce documentation burden. Notably, prospective studies reveal that such multimodal assistance leads to measurable improvements in patient outcomes, including better adherence to treatment plans and follow-up recommendations (66, 67). The closed-loop integration between imaging interpretation and clinical decision support represents a major advance in ophthalmic AI applications.

Besides workflow optimization, multimodal NLP serves as an important safeguard against diagnostic errors through cross-validation of imaging findings with clinical documentation (68). The technology can flag discrepancies in laterality documentation or identify potentially overlooked abnormalities in free-text reports, improving compliance with clinical guidelines.

The clinical value of the systems extends beyond ocular conditions, as evidenced by emerging applications in systemic disease prediction. The eye’s unique anatomical features provide a window to the body’s health, with retinal biomarkers offering insights into cardiovascular and neurological risks (69, 70). Such predictive analytics, when embedded in routine screenings, could facilitate early intervention and primary prevention to stroke, myocardial infarction, dementia, and Parkinson’s disease, positioning ophthalmology as a gateway for holistic healthcare monitoring (14, 71). The synergy of NLP with multimodal data thus not only elevates diagnostic precision but also redefines proactive medicine.

### 3.3 Patient communication and management

Multimodal NLP is revolutionizing patient engagement and chronic disease management in ophthalmology. First, VQA systems empower patients by enabling natural language queries about their diagnostic images (72–74). The AI-powered interfaces can explain pathological findings in lay terms, clarify treatment plans, and even highlight concerning changes in serial images, bridging the gap between technical medical data and patient comprehension. Second, NLP can facilitate structured longitudinal documentation by automatically extracting relevant metrics from free-text clinical notes and imaging reports for the prevention and treatment of chronic conditions like DR or glaucoma (75–77). For instance, in DR management, autonomous AI-driven point-of-care exams have been shown to significantly increase eye exam completion rates (100% vs. 22%, *p* < 0.001) and improve follow-through with eye care providers (64% vs. 22%) in youth with diabetes. The system can support the personalized follow-up scheduling, medication adherence tracking, and automated risk-stratified alerts, ensuring continuity of care while reducing administrative burdens. Third, multilingual support addresses healthcare disparities by providing real-time translation of ophthalmic terminology in patient education materials, informed consent documents, and telemedicine consultations (78–80). For example, the DeepDR-LLM system can generate management recommendations in multilingual settings (English and Chinese) which were comparable in quality to endocrinology residents (66), thereby enhancing diagnostic reliability while reducing physician workload. Particularly valuable in diverse populations, these systems maintain clinical accuracy while adapting to cultural contexts.

### 3.4 Physician assistance and education

Multimodal NLP serves as a powerful clinical copilot in ophthalmology, providing real-time decision support and reducing diagnostic variability. NLP systems like DeepDR-LLM enable primary care physicians to achieve 92.3% accuracy in referable DR detection (vs. 81.0% unassisted), demonstrating how language models can bridge expertise gaps in resource-limited settings (66).

NLP is also transforming medical education by overcoming traditional training limitations (81). The retrieval-augmented NLP framework, large language model-based digital patient (LLMDP) system (82), converts de-identified EHRs into interactive virtual patients, with RCT results showing significant improvements in students’ history-taking skills. The system’s adaptive feedback and free-text dialog capabilities demonstrate NLP’s potential to enhance clinical empathy and prepare trainees for real patient interactions.

For rare ocular diseases where clinical exposure is limited, NLP tools mitigate knowledge disparities by correlating multimodal data to generate evidence-based prompts (83). The EyeFM model exemplifies this through its human-in-the-loop design, where the NLP-driven standardization of reporting terminology not only improved diagnostic accuracy but also served as a real-time educational aid for clinicians across different experience levels (65). By providing consistent, evidence-based diagnostic suggestions, the system helps bridge the gap between expert and non-expert practitioners, ensuring higher quality care and fostering continuous learning in complex diagnostic scenarios.

These NLP applications create a comprehensive support system-assisting real-time clinical decisions while providing scalable training solutions. By linking imaging findings with textual clinical descriptors, NLP serves as a digital mentor that democratizes ophthalmic expertise across healthcare tiers and addresses the long-tail distribution of rare disease cases in education.

## 4 Challenges and limitations

The integration of multimodal NLP into ophthalmic practice presents several critical challenges that must be addressed to ensure clinical validity, ethical deployment, and regulatory compliance. These limitations span technical, ethical, and practical domains, highlighting key areas for future research and development.

### 4.1 Data privacy and security

The use of sensitive clinical text and medical imaging raises substantial privacy concerns (84, 85). While anonymization techniques are standard, such as de-identification of protected health information, multimodal NLP systems must ensure that reconstructed embeddings or joint representations do not inadvertently leak patient-identifiable data.

### 4.2 Domain adaptation and specialized terminology

General-purpose transformer models, such as GPT-4 and BERT, often lack domain-specific knowledge required for accurate ophthalmic applications (86). Fine-tuning on specialized corpora, including clinical notes, imaging reports, and ophthalmology literature, is essential to improve performance on clinical and professional tasks (87, 88). However, the current scarcity of high-quality, labeled ophthalmic datasets remains a bottleneck for further model optimization.

### 4.3 Interpretability and clinician trust

The “black-box” nature of many AI systems poses a significant barrier to clinical applications, which is more obvious in more advanced and complex technologies (89). Physicians need transparent, interpretable, and trustful reasoning behind AI-generated conclusions. Techniques such as attention visualization, concept activation vectors, and hybrid symbolic-neural approaches may help bridge the gap, but deeper validation is required to ensure explanations align with clinical trusts (90, 91).

### 4.4 Data harmonization and label consistency

Discrepancies in labeling protocols, imaging artifacts, and heterogeneous data sources introduce inconsistencies that degrade model performance. These variations extend beyond technical discordance, and the multimodal systems risk compounding biases inherited from both textual and imaging datasets. Demographic biases emerge when training data underrepresent certain populations (92–94). Institutional biases arise from variability in EHR documentation practices or imaging hardware, limiting generalizability across clinical settings (95). Linguistic biases further exacerbate disparities, as the predominance of English-language medical literature marginalizes non-English-speaking populations. Achieving equitable performance requires not only technical standardization but also proactive curation of diverse, representative datasets and rigorous bias audits throughout the model lifecycle.

### 4.5 Regulatory and clinical validation hurdles

The integration of multimodal NLP systems in ophthalmology encounters significant regulatory challenges, particularly in meeting the rigorous standards set by agencies like the FDA and NMPA (96, 97). These systems are required not only to demonstrate high accuracy but also provide clear explanations of how they combine and interpret both textual and imaging data to reach clinical conclusions. Furthermore, ensuring consistent performance across diverse clinical settings, with variations in imaging equipment and HER systems, adds another layer of complexity. Real-world validation through pragmatic clinical trials will be essential to establish the reliability and generalizability of these AI-driven solutions in routine ophthalmic practice (98).

## 5 Future directions

The rapid advancement of multimodal NLP in ophthalmology is paving the way for transformative clinical applications, addressing current limitations while unlocking new possibilities at the intersection of language and medical imaging. Several promising research directions are emerging, each with the potential to reshape diagnostic and therapeutic practices.

### 5.1 Privacy-preserving federated learning

The development of multimodal models often requires large-scale datasets, yet privacy regulations restrict centralized data aggregation (99, 100). Federated learning offers a decentralized solution by training models across hospitals while keeping raw data localized (101–103). Techniques such as differential privacy (DP) can prevent the reconstruction of sensitive patient information from model gradients (104). Furthermore, personalized aggregation strategies can address domain shift caused by institutional variations in imaging equipment or documentation styles, ensuring model generalizability without compromising data security.

### 5.2 Few-shot learning for domain-specific adaptation

A major challenge in ophthalmic AI is the limited availability of high-quality annotated datasets, given the specialized nature of clinical text and imaging. Few-shot learning techniques, such as prompt-based fine-tuning of LLMs and contrastive vision-language pretraining, could enable models to generalize effectively even with sparse labeled examples (105, 106). The approaches leverage semantically rich embeddings to interpret rare retinal conditions using minimal annotated reports. Meta-learning strategies may further enhance adaptability across healthcare systems with disparate documentation standards, ensuring robust performance despite data variability (107, 108).

### 5.3 Real-time interactive systems for clinical workflows

Future multimodal NLP systems are expected to evolve from static analysis tools to dynamic, real-time assistants integrated into clinical workflows. Potential applications include intraoperative voice-to-text documentation, where AI converts surgical narration into structured operative notes synchronized with microscope video (109, 110). Another promising avenue is context-aware clinical decision support, which retrieves relevant literature or prior imaging studies by parsing live physician-patient dialogs. Achieving these capabilities will require advancements in speech recognition, low-latency processing, and seamless interoperability with electronic health records.

### 5.4 Multimodal foundation models for ophthalmology

A promising future direction involves the development of ophthalmology-specific foundation models pretrained on diverse multimodal datasets. These models could integrate cross-modal corpora, aligning text-image pairs from published literature, EHRs, and public datasets. Such foundation models could serve as versatile platforms for transfer learning, reducing reliance on task-specific annotations and accelerating deployment across various clinical applications.

### 5.5 Ethical frameworks for generative AI deployment

As generative AI tools become more prevalent in ophthalmology, there is an urgent need for standardized ethical guidelines (111). Key considerations include accountability in verifying AI-generated diagnostic suggestions to prevent hallucinated references to non-existent imaging findings. Additionally, clear boundaries must be established regarding AI autonomy in drafting clinical correspondence, ensuring physician oversight where necessary. Transparency in patient communication about AI involvement in report generation or telemedicine consultations is also critical. Professional societies should collaborate with ethicists to develop policies that balance innovation with patient safety and trust (112).

## 6 Conclusion

Multimodal NLP serves as a critical bridge between ophthalmology’s rich textual data and complex visual information, fundamentally transforming how we diagnose and manage eye diseases. This powerful synergy of language and imaging AI enables more accurate interpretations, efficient workflows, and personalized treatment approaches. To fully realize this potential, the field must prioritize developing comprehensive standardized datasets that align clinical narratives with corresponding medical images. The path forward requires close collaboration between ophthalmologists, AI specialists, and healthcare administrators to address technical challenges and ethical considerations. As these technologies mature, they promise to not just assist but elevate ophthalmic practice, ultimately delivering better outcomes for patients worldwide.

## References

[1] Bedi S Liu Y Orr-Ewing L Dash D Koyejo S Callahan A . Testing and evaluation of health care applications of large language models: a systematic review. JAMA. (2025) 333:319–28. doi: 10.1001/jama.2024.21700, 39405325 PMC11480901

[2] Haug CJ Drazen JM. Artificial intelligence and machine learning in clinical medicine, 2023. N Engl J Med. (2023) 388:1201–8. doi: 10.1056/NEJMra2302038, 36988595

[3] Thirunavukarasu AJ Ting DSJ Elangovan K Gutierrez L Tan TF Ting DSW. Large language models in medicine. Nat Med. (2023) 29:1930–40. doi: 10.1038/s41591-023-02448-8, 37460753

[4] Ting DSW Lin H Ruamviboonsuk P Wong TY Sim DA. Artificial intelligence, the internet of things, and virtual clinics: ophthalmology at the digital translation forefront. Lancet Digit Health. (2020) 2:e8–9. doi: 10.1016/s2589-7500(19)30217-1, 33328040

[5] Betzler BK Chen H Cheng CY Lee CS Ning G Song SJ . Large language models and their impact in ophthalmology. Lancet Digit Health. (2023) 5:e917–24. doi: 10.1016/s2589-7500(23)00201-7, 38000875 PMC11003328

[6] Liu F Zhou H Wang K Yu Y Gao Y Sun Z . Metagp: a generative foundation model integrating electronic health records and multimodal imaging for addressing unmet clinical needs. Cell Rep Med. (2025) 6:102056. doi: 10.1016/j.xcrm.2025.102056, 40187356 PMC12047458

[7] Viggiano P Boscia G Sadeghi E Cheung G Borrelli E Alessio G . Pachychoroid disease Spectrum: how multimodal imaging and Oct angiography have improved our knowledge. Prog Retin Eye Res. (2025) 107:101372. doi: 10.1016/j.preteyeres.2025.101372, 40414595

[8] Flaxel CJ Adelman RA Bailey ST Fawzi A Lim JI Vemulakonda GA . Diabetic retinopathy preferred practice pattern®. Ophthalmology. (2020) 127:P66–P145. doi: 10.1016/j.ophtha.2019.09.025 31757498

[9] Pazos M Traverso CE Viswanathan A. European Glaucoma society - terminology and guidelines for Glaucoma, 6th edition. Br J Ophthalmol. (2025) 109:1–212. doi: 10.1136/bjophthalmol-2025-egsguidelines 41026937

[10] Holler J Levinson SC. Multimodal language processing in human communication. Trends Cogn Sci. (2019) 23:639–52. doi: 10.1016/j.tics.2019.05.006, 31235320

[11] Strotzer QD Nieberle F Kupke LS Napodano G Muertz AK Meiler S . Toward foundation models in radiology? Quantitative assessment of Gpt-4v's multimodal and multianatomic region capabilities. Radiology. (2024) 313:e240955. doi: 10.1148/radiol.240955, 39589253

[12] Zhang Z Chen K Wang R Utiyama M Sumita E Li Z . Universal multimodal representation for language understanding. IEEE Trans Pattern Anal Mach Intell. (2023) 45:1–18. doi: 10.1109/tpami.2023.3234170, 37018264

[13] Jiachu D Luo L Xie M Xie X Guo J Ye H . A Meta-learning approach for classifying multimodal retinal images of retinal vein occlusion with limited data. Transl Vis Sci Technol. (2024) 13:22. doi: 10.1167/tvst.13.9.22, 39297809 PMC11421671

[14] Shi D Zhang W Yang J Huang S Chen X Xu P . A multimodal visual-language foundation model for computational ophthalmology. NPJ Digit Med. (2025) 8:381. doi: 10.1038/s41746-025-01772-2, 40542189 PMC12181238

[15] Rojas-Carabali W Agrawal R Gutierrez-Sinisterra L Baxter SL Cifuentes-González C Wei YC . Natural language processing in medicine and ophthalmology: a review for the 21st-century clinician. Asia Pac J Ophthalmol. (2024) 13:100084. doi: 10.1016/j.apjo.2024.100084, 39059557 PMC11919464

[16] Tan TF Thirunavukarasu AJ Campbell JP Keane PA Pasquale LR Abramoff MD . Generative artificial intelligence through Chatgpt and other large language models in ophthalmology: clinical applications and challenges. Ophthalmol Sci. (2023) 3:100394. doi: 10.1016/j.xops.2023.100394, 37885755 PMC10598525

[17] Oudeyer PY. Autonomous development and learning in artificial intelligence and robotics: scaling up deep learning to human-like learning. Behav Brain Sci. (2017) 40:e275. doi: 10.1017/s0140525x17000243, 29342696

[18] Georgevici AI Terblanche M. Neural networks and deep learning: a brief introduction. Intensive Care Med. (2019) 45:712–4. doi: 10.1007/s00134-019-05537-w, 30725133

[19] Theodosiou AA Read RC. Artificial intelligence, machine learning and deep learning: potential resources for the infection clinician. J Infect. (2023) 87:287–94. doi: 10.1016/j.jinf.2023.07.006, 37468046

[20] Balyen L Peto T. Promising artificial intelligence-machine learning-deep learning algorithms in ophthalmology. Asia Pac J Ophthalmol. (2019) 8:264–72. doi: 10.22608/apo.2018479 31149787

[21] Jowsey T Stokes-Parish J Singleton R Todorovic M. Medical education empowered by generative artificial intelligence large language models. Trends Mol Med. (2023) 29:971–3. doi: 10.1016/j.molmed.2023.08.012, 37718142

[22] Yi PH Haver HL Jeudy JJ Kim W Kitamura FC Oluyemi ET . Best practices for the safe use of large language models and other generative AI in radiology. Radiology. (2025) 316:e241516. doi: 10.1148/radiol.241516, 40985835 PMC12501631

[23] Muller L Churchland PS Sejnowski TJ. Transformers and cortical waves: encoders for pulling in context across time. Trends Neurosci. (2024) 47:788–802. doi: 10.1016/j.tins.2024.08.006, 39341729 PMC11936488

[24] Kitamura FC Farina E Mazuco Rodriguez JP Moy L Prevedello LM. Texts are more than notes, they are data: a glimpse into how machines understand text. Radiology. (2025) 316:e243217. doi: 10.1148/radiol.243217, 40892454

[25] Guo R Wei J Sun L Yu B Chang G Liu D . A survey on advancements in image-text multimodal models: from general techniques to biomedical implementations. Comput Biol Med. (2024) 178:108709. doi: 10.1016/j.compbiomed.2024.108709, 38878398

[26] Lin Z Zhang D Shi D Xu R Tao Q Wu L . Contrastive pre-training and linear interaction attention-based transformer for universal medical reports generation. J Biomed Inform. (2023) 138:104281. doi: 10.1016/j.jbi.2023.104281, 36638935

[27] Song X Chao H Xu X Guo H Xu S Turkbey B . Cross-modal attention for multi-modal image registration. Med Image Anal. (2022) 82:102612. doi: 10.1016/j.media.2022.102612, 36126402 PMC9588729

[28] Oghbaie M Araújo T Schmidt-Erfurth U Bogunović H. Vlfatrollout: fully transformer-based classifier for retinal Oct volumes. Comput Med Imaging Graph. (2024) 118:102452. doi: 10.1016/j.compmedimag.2024.102452, 39489098

[29] Liu Z Hu Y Qiu Z Niu Y Zhou D Li X . Cross-modal attention network for retinal disease classification based on multi-modal images. Biomed Opt Express. (2024) 15:3699–714. doi: 10.1364/boe.516764, 38867787 PMC11166426

[30] Chen Q Hu Y Peng X Xie Q Jin Q Gilson A . Benchmarking large language models for biomedical natural language processing applications and recommendations. Nat Commun. (2025) 16:3280. doi: 10.1038/s41467-025-56989-2, 40188094 PMC11972378

[31] Haghighi T Gholami S Sokol JT Kishnani E Ahsaniyan A Rahmanian H . Eye-Llama, an in-domain large language model for ophthalmology. iScience. (2025) 28:112984. doi: 10.1016/j.isci.2025.112984, 40697409 PMC12281063

[32] Hillenmayer A Lofi B Langhans S Elhardt C Wolf A Wertheimer CM. Neural network for natural language processing to determine treatment urgency in an ophthalmology emergency department. Br J Ophthalmol. (2025) 110:17–24. doi: 10.1136/bjo-2025-327824, 40819882 PMC12772578

[33] Akhlaq A Liu TYA. Text-to-video generation-a cutting-edge generative Ai application. JAMA Ophthalmol. (2025) 143:632–3. doi: 10.1001/jamaophthalmol.2025.2023, 40569630

[34] Antaki F Touma S Milad D El-Khoury J Duval R. Evaluating the performance of Chatgpt in ophthalmology: An analysis of its successes and shortcomings. Ophthalmol Sci. (2023) 3:100324. doi: 10.1016/j.xops.2023.100324, 37334036 PMC10272508

[35] Grauslund J. Diabetic retinopathy screening in the emerging era of artificial intelligence. Diabetologia. (2022) 65:1415–23. doi: 10.1007/s00125-022-05727-0, 35639120

[36] Gulshan V Peng L Coram M Stumpe MC Wu D Narayanaswamy A . Development and validation of a deep learning algorithm for detection of diabetic retinopathy in retinal fundus photographs. JAMA. (2016) 316:2402–10. doi: 10.1001/jama.2016.17216, 27898976

[37] Fan R Bowd C Brye N Christopher M Weinreb RN Kriegman DJ . One-vote veto: semi-supervised learning for low-shot Glaucoma diagnosis. IEEE Trans Med Imaging. (2023) 42:3764–78. doi: 10.1109/tmi.2023.3307689, 37610903 PMC11214580

[38] Won YK Kim CH Jeon J Cha J Lim DH. Deep learning by vision transformer to classify bacterial and fungal keratitis using different types of anterior segment images. Comput Biol Med. (2025) 190:109976. doi: 10.1016/j.compbiomed.2025.109976, 40107025

[39] Playout C Duval R Boucher MC Cheriet F. Focused attention in transformers for interpretable classification of retinal images. Med Image Anal. (2022) 82:102608. doi: 10.1016/j.media.2022.102608, 36150271

[40] Hou K Bradley C Herbert P Johnson C Wall M Ramulu PY . Predicting visual field worsening with longitudinal Oct data using a gated transformer network. Ophthalmology. (2023) 130:854–62. doi: 10.1016/j.ophtha.2023.03.019, 37003520 PMC10524436

[41] Peng Y Lin A Wang M Lin T Liu L Wu J . Enhancing Ai reliability: a foundation model with uncertainty estimation for optical coherence tomography-based retinal disease diagnosis. Cell Rep Med. (2025) 6:101876. doi: 10.1016/j.xcrm.2024.101876, 39706192 PMC11866418

[42] Brown EE Guy AA Holroyd NA Sweeney PW Gourmet L Coleman H . Physics-informed deep generative learning for quantitative assessment of the retina. Nat Commun. (2024) 15:6859. doi: 10.1038/s41467-024-50911-y, 39127778 PMC11316734

[43] Ait Hammou B Duval R Boucher MC Cheriet F. Dtg: dual transformers-based generative adversarial networks for retinal 2d/3d Oct image classification. Med Image Anal. (2026) 109:103915. doi: 10.1016/j.media.2025.103915, 41518764

[44] Phipps B Hadoux X Sheng B Campbell JP Liu TYA Keane PA . Ai image generation Technology in Ophthalmology: use, misuse and future applications. Prog Retin Eye Res. (2025) 106:101353. doi: 10.1016/j.preteyeres.2025.101353, 40107410

[45] Lu MY Chen B Williamson DFK Chen RJ Liang I Ding T . A visual-language foundation model for computational pathology. Nat Med. (2024) 30:863–74. doi: 10.1038/s41591-024-02856-4, 38504017 PMC11384335

[46] Shen C Lian C Zhang W Wang F Zhang J Fan S . Large-vocabulary forensic pathological analyses via prototypical cross-modal contrastive learning. Nat Commun. (2025) 16:6773. doi: 10.1038/s41467-025-62060-x, 40702007 PMC12287307

[47] Zhao Z Liu Y Wu H Wang M Li Y Wang S . Clip in medical imaging: a survey. Med Image Anal. (2025) 102:103551. doi: 10.1016/j.media.2025.103551, 40127590

[48] Wang J Wang K Yu Y Lu Y Xiao W Sun Z . Self-improving generative foundation model for synthetic medical image generation and clinical applications. Nat Med. (2025) 31:609–17. doi: 10.1038/s41591-024-03359-y, 39663467

[49] Wang R Yao Q Jiang Z Lai H He Z Tao X . Ecamp: entity-centered context-aware medical vision language pre-training. Med Image Anal. (2025) 105:103690. doi: 10.1016/j.media.2025.103690, 40602209

[50] Duenias D Nichyporuk B Arbel T Riklin Raviv T. Hyperfusion: a hypernetwork approach to multimodal integration of tabular and medical imaging data for predictive modeling. Med Image Anal. (2025) 102:103503. doi: 10.1016/j.media.2025.103503, 40037055

[51] Huang SC Pareek A Seyyedi S Banerjee I Lungren MP. Fusion of medical imaging and electronic health records using deep learning: a systematic review and implementation guidelines. NPJ Digit Med. (2020) 3:136. doi: 10.1038/s41746-020-00341-z, 33083571 PMC7567861

[52] Sloan P Clatworthy P Simpson E Mirmehdi M. Automated radiology report generation: a review of recent advances. IEEE Rev Biomed Eng. (2025) 18:368–87. doi: 10.1109/rbme.2024.3408456, 38829752

[53] Wang X Figueredo G Li R Zhang WE Chen W Chen X. A survey of deep-learning-based radiology report generation using multimodal inputs. Med Image Anal. (2025) 103:103627. doi: 10.1016/j.media.2025.103627, 40382855

[54] Chen X Zhang W Xu P Zhao Z Zheng Y Shi D . Ffa-Gpt: An automated pipeline for fundus fluorescein angiography interpretation and question-answer. NPJ Digit Med. (2024) 7:111. doi: 10.1038/s41746-024-01101-z, 38702471 PMC11068733

[55] Shao A Liu X Shen W Li Y Wu H Pan X . Generative artificial intelligence for fundus fluorescein angiography interpretation and human expert evaluation. NPJ Digit Med. (2025) 8:396. doi: 10.1038/s41746-025-01759-z, 40603524 PMC12222453

[56] Wu X Wang L Chen R Liu B Zhang W Yang X . Generation of fundus fluorescein angiography videos for health care data sharing. JAMA Ophthalmol. (2025) 143:623–32. doi: 10.1001/jamaophthalmol.2025.1419, 40569610 PMC12203415

[57] Xu S Li L Zhu Y Ruan Z Qu Y Zhou Z . School-level prediction and Management of Myopia in children and adolescents. J Transl Med. (2025) 23:876. doi: 10.1186/s12967-025-06855-y, 40770756 PMC12330041

[58] Wu J Fang H Li F Fu H Lin F Li J . Gamma challenge: Glaucoma grading from multi-modality images. Med Image Anal. (2023) 90:102938. doi: 10.1016/j.media.2023.102938, 37806020

[59] Dixit A Yohannan J Boland MV. Assessing Glaucoma progression using machine learning trained on longitudinal visual field and clinical data. Ophthalmology. (2021) 128:1016–26. doi: 10.1016/j.ophtha.2020.12.020, 33359887 PMC8222148

[60] Yang X Wang S Dong J Dong J Wang M Chua TS. Video moment retrieval with cross-modal neural architecture search. IEEE Trans Image Process. (2022) 31:1204–16. doi: 10.1109/tip.2022.3140611, 35015640

[61] Hu D Jiang Z Shi J Xie F Wu K Tang K . Histopathology language-image representation learning for fine-grained digital pathology cross-modal retrieval. Med Image Anal. (2024) 95:103163. doi: 10.1016/j.media.2024.103163, 38626665

[62] Sengupta PP Dey D Davies RH Duchateau N Yanamala N. Challenges for augmenting intelligence in cardiac imaging. Lancet Digit Health. (2024) 6:e739–48. doi: 10.1016/s2589-7500(24)00142-0, 39214759

[63] Tejani AS Cook TS Hussain M Sippel Schmidt T O'Donnell KP. Integrating and adopting Ai in the radiology workflow: a primer for standards and integrating the healthcare enterprise (Ihe) profiles. Radiology. (2024) 311:e232653. doi: 10.1148/radiol.232653 38888474 PMC11208735

[64] Xu P Chen X Zhao Z Shi D. Unveiling the clinical Incapabilities: a benchmarking study of Gpt-4v(Ision) for ophthalmic multimodal image analysis. Br J Ophthalmol. (2024) 108:1384–9. doi: 10.1136/bjo-2023-325054, 38789133

[65] Wu Y Qian B Li T Qin Y Guan Z Chen T . An Eyecare foundation model for clinical assistance: a randomized controlled trial. Nat Med. (2025) 31:3404–13. doi: 10.1038/s41591-025-03900-7, 40877476

[66] Li J Guan Z Wang J Cheung CY Zheng Y Lim LL . Integrated image-based deep learning and language models for primary diabetes care. Nat Med. (2024) 30:2886–96. doi: 10.1038/s41591-024-03139-8, 39030266 PMC11485246

[67] Pearce FJ Cruz Rivera S Liu X Manna E Denniston AK Calvert MJ. The role of patient-reported outcome measures in trials of artificial intelligence health technologies: a systematic evaluation of clinicaltrials.Gov records (1997-2022). Lancet Digit Health. (1997) 5:e160–7. doi: 10.1016/s2589-7500(22)00249-7, 36828608

[68] Sun C Teichman K Zhou Y Critelli B Nauheim D Keir G . Generative large language models trained for detecting errors in radiology reports. Radiology. (2025) 315:e242575. doi: 10.1148/radiol.242575, 40392090 PMC12130665

[69] Yusufu M Friedman DS Kang M Padhye A Shang X Zhang L . Retinal vascular fingerprints predict incident stroke: findings from the Uk biobank cohort study. Heart. (2025) 111:306–13. doi: 10.1136/heartjnl-2024-324705, 39805634

[70] Gupta K Reddy S. Heart, eye, and artificial intelligence: a review. Cardiol Res. (2021) 12:132–9. doi: 10.14740/cr1179, 34046105 PMC8139752

[71] Lee TK Kim SY Choi HJ Choe EK Sohn KA. Vision transformer based interpretable metabolic syndrome classification using retinal images. NPJ Digit Med. (2025) 8:205. doi: 10.1038/s41746-025-01588-0, 40216912 PMC11992118

[72] Peng L Yang Y Wang Z Huang Z Shen HT. Mra-net: improving Vqa via multi-modal relation attention network. IEEE Trans Pattern Anal Mach Intell. (2022) 44:318–29. doi: 10.1109/tpami.2020.3004830, 32750794

[73] Künzel S Munz-Körner T Tilli P Schäfer N Vidyapu S Thang Vu N . Visual explainable artificial intelligence for graph-based visual question answering and scene graph curation. Vis Comput Ind Biomed Art. (2025) 8:9. doi: 10.1186/s42492-025-00185-y, 40192956 PMC11977082

[74] Gao C Zhu Q Wang P Li H Liu Y Hengel AVD . Structured multimodal attentions for Textvqa. IEEE Trans Pattern Anal Mach Intell. (2022) 44:9603–14. doi: 10.1109/tpami.2021.3132034, 34855584

[75] Rajalakshmi R PramodKumar TA Naziyagulnaaz AS Anjana RM Raman R Manikandan S . Leveraging artificial intelligence for diabetic retinopathy screening and management: history and current advances. Semin Ophthalmol. (2025) 40:719–26. doi: 10.1080/08820538.2024.2432902, 39580713

[76] Wolf RM Channa R Liu TYA Zehra A Bromberger L Patel D . Autonomous artificial intelligence increases screening and follow-up for diabetic retinopathy in youth: the access randomized control trial. Nat Commun. (2024) 15:421. doi: 10.1038/s41467-023-44676-z, 38212308 PMC10784572

[77] Li F Wang D Yang Z Zhang Y Jiang J Liu X . The Ai revolution in Glaucoma: bridging challenges with opportunities. Prog Retin Eye Res. (2024) 103:101291. doi: 10.1016/j.preteyeres.2024.101291, 39186968

[78] Lee YQ Chen CT Chen CC Lee CH Chen P Wu CS . Unlocking the secrets behind advanced artificial intelligence language models in Deidentifying Chinese-English mixed clinical text: development and validation study. J Med Internet Res. (2024) 26:e48443. doi: 10.2196/48443, 38271060 PMC10853853

[79] Qiu P Wu C Zhang X Lin W Wang H Zhang Y . Towards building multilingual language model for medicine. Nat Commun. (2024) 15:8384. doi: 10.1038/s41467-024-52417-z, 39333468 PMC11436924

[80] Menz BD Modi ND Abuhelwa AY Ruanglertboon W Vitry A Gao Y . Generative Ai Chatbots for reliable Cancer information: evaluating web-search, multilingual, and reference capabilities of emerging large language models. Eur J Cancer. (2025) 218:115274. doi: 10.1016/j.ejca.2025.115274, 39922126

[81] Ning Y Ong JCL Cheng H Wang H Ting DSW Tham YC . How can artificial intelligence transform the training of medical students and physicians? Lancet Digit Health. (2025) 7:100900. doi: 10.1016/j.landig.2025.100900, 41047321

[82] Luo MJ Bi S Pang J Liu L Tsui CK Lai Y . A large language model digital patient system enhances ophthalmology history taking skills. NPJ Digit Med. (2025) 8:502. doi: 10.1038/s41746-025-01841-6, 40760042 PMC12322286

[83] Hirsch MC Ronicke S Krusche M Wagner AD. Rare diseases 2030: how augmented Ai will support diagnosis and treatment of rare diseases in the future. Ann Rheum Dis. (2020) 79:740–3. doi: 10.1136/annrheumdis-2020-217125, 32209541 PMC7286047

[84] Price WN 2nd Cohen IG. Privacy in the age of medical big data. Nat Med. (2019) 25:37–43. doi: 10.1038/s41591-018-0272-7, 30617331 PMC6376961

[85] Zhou J Huang C Gao X. Patient privacy in Ai-driven omics methods. Trends Genet. (2024) 40:383–6. doi: 10.1016/j.tig.2024.03.004, 38637270

[86] Bian C Yuan C Wang J Li M Yang X Yu S . Uncertainty-aware domain alignment for anatomical structure segmentation. Med Image Anal. (2020) 64:101732. doi: 10.1016/j.media.2020.101732, 32580058

[87] Li W Zhang Y Zhou H Yang W Xie Z He Y. Clms: bridging domain gaps in medical imaging segmentation with source-free continual learning for robust knowledge transfer and adaptation. Med Image Anal. (2025) 100:103404. doi: 10.1016/j.media.2024.103404, 39616943

[88] Li Z Wang Y Qiang W Wu X Zhang Y Gu Y . A domain-specific Pretrained model for detecting malignant and premalignant ocular surface tumors: a multicenter model development and evaluation study. Research. (2025) 8:0711. doi: 10.34133/research.0711, 40421109 PMC12104561

[89] Castelvecchi D. Can we open the black box of Ai? Nature. (2016) 538:20–3. doi: 10.1038/538020a, 27708329

[90] Collins GS. Making the black box more transparent: improving the reporting of artificial intelligence studies in healthcare. BMJ. (2024) 385:q832. doi: 10.1136/bmj.q832, 38626954

[91] Wen C Ye M Li H Chen T Xiao X. Concept-based lesion aware transformer for interpretable retinal disease diagnosis. IEEE Trans Med Imaging. (2025) 44:57–68. doi: 10.1109/tmi.2024.3429148, 39012729

[92] Zack T Lehman E Suzgun M Rodriguez JA Celi LA Gichoya J . Assessing the potential of Gpt-4 to perpetuate racial and gender biases in health care: a model evaluation study. Lancet Digit Health. (2024) 6:e12–22. doi: 10.1016/s2589-7500(23)00225-x, 38123252

[93] Mihan A Pandey A Van Spall HG. Mitigating the risk of artificial intelligence bias in cardiovascular care. Lancet Digit Health. (2024) 6:e749–54. doi: 10.1016/s2589-7500(24)00155-9, 39214762

[94] Hu T Kyrychenko Y Rathje S Collier N van der Linden S Roozenbeek J. Generative language models exhibit social identity biases. Nat Comput Sci. (2025) 5:65–75. doi: 10.1038/s43588-024-00741-1, 39668254 PMC11774750

[95] Omar M Soffer S Agbareia R Bragazzi NL Apakama DU Horowitz CR . Sociodemographic biases in medical decision making by large language models. Nat Med. (2025) 31:1873–81. doi: 10.1038/s41591-025-03626-6, 40195448

[96] Ong JCL Chang SY William W Butte AJ Shah NH Chew LST . Ethical and regulatory challenges of large language models in medicine. Lancet Digit Health. (2024) 6:e428–32. doi: 10.1016/s2589-7500(24)00061-x, 38658283

[97] Ning Y Teixayavong S Shang Y Savulescu J Nagaraj V Miao D . Generative artificial intelligence and ethical considerations in health care: a scoping review and ethics checklist. Lancet Digit Health. (2024) 6:e848–56. doi: 10.1016/s2589-7500(24)00143-2, 39294061 PMC11542614

[98] Li Z Wang L Wu X Jiang J Qiang W Xie H . Artificial intelligence in ophthalmology: the path to the real-world clinic. Cell Rep Med. (2023) 4:101095. doi: 10.1016/j.xcrm.2023.101095, 37385253 PMC10394169

[99] Arora A Wagner SK Carpenter R Jena R Keane PA. The urgent need to accelerate synthetic data privacy frameworks for medical research. Lancet Digit Health. (2025) 7:e157–60. doi: 10.1016/s2589-7500(24)00196-1, 39603900

[100] Suri A Summers RM. Privacy, please: safeguarding medical data in imaging Ai using differential privacy techniques. Radiol Artif Intell. (2024) 6:e230560. doi: 10.1148/ryai.230560, 38231038 PMC10831504

[101] Teo ZL Zhang X Yang Y Jin L Zhang C Poh SSJ . Privacy-preserving technology using federated learning and blockchain in protecting against adversarial attacks for retinal imaging. Ophthalmology. (2025) 132:484–94. doi: 10.1016/j.ophtha.2024.10.017 39424148

[102] Koutsoubis N Waqas A Yilmaz Y Ramachandran RP Schabath MB Rasool G. Privacy-preserving federated learning and uncertainty quantification in medical imaging. Radiol Artif Intell. (2025) 7:e240637. doi: 10.1148/ryai.240637, 40366260 PMC12319697

[103] Zhou J Chen S Wu Y Li H Zhang B Zhou L . Ppml-omics: a privacy-preserving federated machine learning method protects patients’ privacy in Omic data. Sci Adv. (2024) 10:eadh8601. doi: 10.1126/sciadv.adh8601, 38295178 PMC10830108

[104] Zhang L Xu J Sivaraman A Deborah Lazarus J Sharma PK Pandi V. A two-stage differential privacy scheme for federated learning based on edge intelligence. IEEE J Biomed Health Inform. (2024) 28:3349–60. doi: 10.1109/jbhi.2023.3306425, 37594867

[105] Xue H An Y Qin Y Li W Wu Y Che Y . Toward few-shot learning in the open world: a review and beyond. IEEE Trans Pattern Anal Mach Intell. (2025) 47:10420–40. doi: 10.1109/tpami.2025.3594686, 40748790

[106] Quellec G Lamard M Conze PH Massin P Cochener B. Automatic detection of rare pathologies in fundus photographs using few-shot learning. Med Image Anal. (2020) 61:101660. doi: 10.1016/j.media.2020.101660, 32028213

[107] Hospedales T Antoniou A Micaelli P Storkey A. Meta-learning in neural networks: a survey. IEEE Trans Pattern Anal Mach Intell. (2022) 44:1–5169. doi: 10.1109/tpami.2021.3079209, 33974543

[108] Gao M Jiang H Zhu L Jiang Z Geng M Ren Q . Discriminative ensemble Meta-learning with co-regularization for rare fundus diseases diagnosis. Med Image Anal. (2023) 89:102884. doi: 10.1016/j.media.2023.102884, 37459674

[109] Lux TJ Saßmannshausen Z Kafetzis I Sodmann P Herold K Sudarevic B . Assisted documentation as a new focus for artificial intelligence in endoscopy: the precedent of reliable withdrawal time and image reporting. Endoscopy. (2023) 55:1118–23. doi: 10.1055/a-2122-1671, 37399844 PMC11321719

[110] Pan J Xia J Jiang B Zhang H Zhang H Li ZS . Real-time identification of gastric lesions and anatomical landmarks by artificial intelligence during magnetically controlled capsule endoscopy. Endoscopy. (2022) 54:E622–e623. doi: 10.1055/a-1724-6958, 35081639

[111] Vasey B Nagendran M Campbell B Clifton DA Collins GS Denaxas S . Reporting guideline for the early stage clinical evaluation of decision support systems driven by artificial intelligence: decide-AI. BMJ. (2022) 377:e070904. doi: 10.1136/bmj-2022-070904, 35584845 PMC9116198

[112] Panteli D Adib K Buttigieg S Goiana-da-Silva F Ladewig K Azzopardi-Muscat N . Artificial intelligence in public health: promises, challenges, and an agenda for policy makers and public health institutions. Lancet Public Health. (2025) 10:e428–32. doi: 10.1016/s2468-2667(25)00036-2, 40031938 PMC12040707

